# Sarcopenia changes and incident type 2 diabetes mellitus: a prospective analysis of the CHARLS cohort

**DOI:** 10.1186/s12877-026-07169-4

**Published:** 2026-03-13

**Authors:** Lingli Gao, Yan Chen, Shuoshuo Su, Yixun Chen, Lidian Chen, Jing Gao, Xiaodong Feng

**Affiliations:** 1 The First Affiliated Hospital of Henan University of Chinese Medicine, zhengzhou, China; 2 School of Rehabilitation Sciences of Henan, University of Chinese Medicine, zhengzhou, China

**Keywords:** Sarcopenia, Type 2 diabetes mellitus, Dynamic nature, Epidemiology

## Abstract

**Background:**

Emerging evidence suggests that sarcopenia may be a significant risk factor for Type 2 Diabetes Mellitus (T2DM). However, existing research has predominantly relied on single baseline assessments, leaving the impact of its dynamic progression over time unclear. This study aimed to prospectively evaluate how transitions in sarcopenia status influence the incidence of T2DM.

**Methods:**

We analyzed data from the China Health and Retirement Longitudinal Study (CHARLS). Sarcopenia was defined according to the 2019 AWGS criteria and classified as non-sarcopenia, possible sarcopenia, or sarcopenia. Changes in status were determined by comparing classifications at baseline (2011year) and a two-year follow-up(2013year). Incident T2DM was identified through elevated fasting plasma glucose, random plasma glucose, HbA1c levels, or self-reported physician diagnosis. Hazard ratios (HRs) and 95% confidence intervals (CIs) were estimated using Cox regression models, adjusted for multiple potential confounders.

**Results:**

Among 3,673 eligible participants (51.3% male; mean age 68.21 years), regression analysis revealed that recovery from sarcopenia to a non-sarcopenic state was associated with a significantly lower risk of T2DM (HR = 0.61, 95% CI: 0.40–0.93) compared to those who remained sarcopenic. While progression to a worse sarcopenia state showed a trend towards higher T2DM risk, this association was not statistically significant. Subgroup analyses indicated that the protective association of sarcopenia reversal was significant in men (HR = 0.43, 95% CI: 0.24–0.78) and participants aged ≥ 65 years (HR = 0.60, 95% CI: 0.37–0.99), but not in women or those under 65.

**Conclusions:**

The longitudinal trajectory of sarcopenia status is associated with the risk of developing T2DM. Notably, improvement from sarcopenia is linked to a reduced risk, whereas progression may indicate an elevated risk, underscoring the potential clinical value of monitoring and managing sarcopenia dynamics.

**Supplementary Information:**

The online version contains supplementary material available at 10.1186/s12877-026-07169-4.

## Background

Sarcopenia, a clinical syndrome characterized by the progressive and generalized loss of skeletal muscle mass and strength associated with aging, is recognized as one of the key manifestations of geriatric syndromes. It imposes a substantial economic and social burden due to its adverse health outcomes and the consequent need for increased healthcare utilization and long-term support [1, 2].

Epidemiological evidence indicates that approximately 10% to 16% of older adults worldwide are affected by sarcopenia, with prevalence rates in Asian populations ranging from 5.5% to 25.7%. In China, the prevalence among community-dwelling older adults varies between 8.9% and 38.8%, rising sharply to 67.1% in individuals aged 80 years and older [3, 4]. In recent years, sarcopenia has been increasingly identified as a significant comorbidity in individuals with diabetes. As the prevalence and incidence of diabetes rise markedly with advancing age, the coexistence of sarcopenia and diabetes has become a growing concern. This bidirectional relationship has drawn increasing attention from researchers and clinicians in the field of diabetes care [5]. A more comprehensive understanding of the interplay between sarcopenia and diabetes may offer novel insights into the prevention and management of diabetes in aging populations.

Prior research has indicated that individuals with possible or confirmed sarcopenia face a higher likelihood of developing diabetes. Nevertheless, the majority of these studies have concentrated on the initial presence of sarcopenia, neglecting the evolving nature of sarcopenia throughout the study duration [6, 7]. In contrast to single-timepoint assessments, longitudinal monitoring of sarcopenia status enables a more comprehensive understanding of underlying biological relationships, including the potential protective effect of sarcopenia reversal on the development of T2DM. Importantly, accumulating evidence suggests that sarcopenia is a modifiable condition, with reversibility achievable through targeted interventions such as nutritional supplementation and structured exercise programs [8–10]. Consequently, assessing the incidence of new-onset T2DM among individuals who recover from sarcopenia may provide critical evidence to support the integration of sarcopenia-directed interventions into clinical strategies for diabetes prevention and management. Given these considerations, investigating the association between changes in sarcopenia status over time and the risk of incident diabetes holds substantial clinical relevance and public health significance.

Currently, the impact of dynamic changes in sarcopenia status on the incidence of T2DM remains unclear. This study leveraged prospective cohort data from the CHARLS to investigate the association between longitudinal changes in sarcopenia status and the risk of developing T2DM.

## Methods

### Study design and population

The current study utilized data from the CHARLS. The first wave of CHARLS (2011) was designated as the baseline, and the second wave (2013) served as the follow-up assessment. These two waves were used to evaluate dynamic changes in sarcopenia status, while subsequent follow-up waves were employed to track health outcomes up to the fifth wave (2020) [11].

**Fig. 1:**
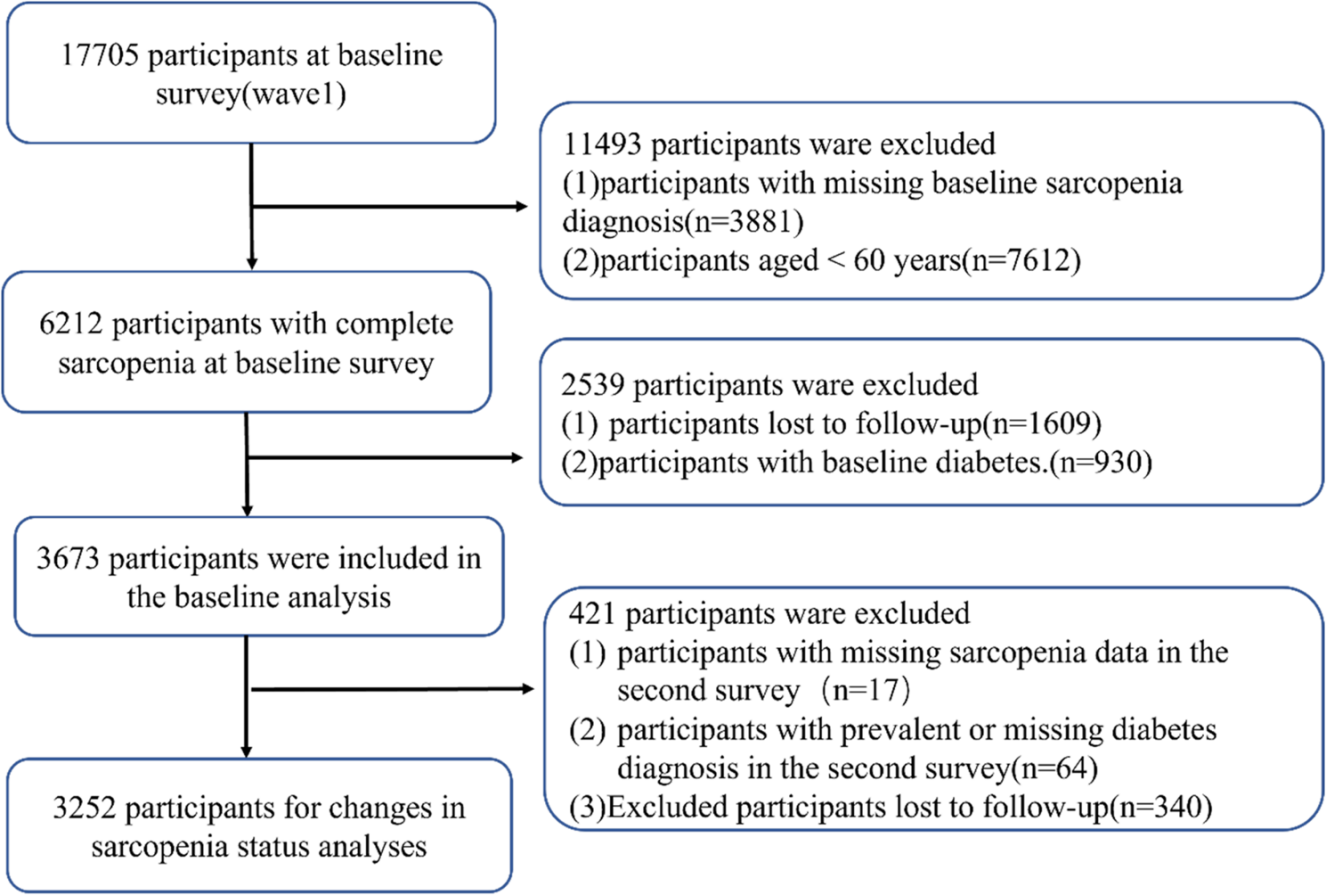
Flow diagram of the study participants

The inclusion criteria for the baseline analysis were as follows: (1) having data on sarcopenia status in the 2011 CHARLS survey; (2) being aged 60 years or older in 2011; (3) having no history of diabetes in 2011; and (4) not being lost to follow-up in the 2013 survey. For the analysis of dynamic changes in sarcopenia status, additional inclusion criteria were set: (5) having data on sarcopenia status in the 2013 CHARLS survey; (6) having no history of diabetes in 2013; and (7) not experiencing loss to follow-up. Among the initial 17,705 CHARLS participants, 3,881 individuals were excluded due to missing data on sarcopenia status. Additionally, 7,612 individuals under the age of 60 were excluded. Furthermore, 1,609 participants who were lost to follow-up in 2013 and 930 participants with a confirmed diagnosis of diabetes at baseline were also excluded. Consequently, 3,673 eligible participants were included in the baseline sarcopenia status analysis. For the longitudinal analysis of sarcopenia dynamics, an additional 421 individuals were excluded based on the aforementioned criteria, resulting in a final sample of 3,252 participants for the analysis (Fig. 1).

### Assessment of sarcopenia status

This study assessed sarcopenia using the 2019 Asian Working Group for Sarcopenia (AWGS) algorithm, which evaluates muscle strength, mass, and physical performance [3]. Sarcopenia was defined as the presence of both low muscle mass and low muscle strength or low physical performance, while low muscle strength or low physical performance alone was classified as possible sarcopenia. If none of the above conditions are met, it is classified as non-sarcopenia (Chen et al., 2020).

Muscle strength was measured by grip strength. Participants were instructed to squeeze a dynamometer as hard as possible, and the maximum value from either hand was used for analysis. Based on AWGS 2019 criteria, low grip strength was defined as < 28 kg for men and < 18 kg for women [12].

ASM was estimated using validated anthropometric equations for the Chinese population [13, 14], and the validity of this estimation model has been confirmed in multiple studies (Y. Liu et al., 2024; Xiong et al., 2023). Low muscle mass was defined as the lowest 20% of ASM/height² values, stratified by sex [13, 15]. The specific cut-off values applied were: for men, < 7.00 kg/m² in both 2011 and 2013; for women, < 5.26 kg/m² in 2011 and < 5.29 kg/m² in 2013.

Physical performance was assessed via gait speed and the five-chair stand test, following procedures described in previous CHARLS studies [16]. According to the AWGS 2019 diagnostic criteria, low physical performance is defined as a gait speed below 1.0 m/s or a completion time of 12 s or longer on the five-time sit-to-stand test [3].The definitions of each component of sarcopenia in the CHARLS cohort have been elaborated in detail in previous studies [3]. Based on these criteria, participants were categorized into three groups at baseline: non-sarcopenia (*n* = 1,331), possible sarcopenia (*n* = 1,431), and sarcopenia (*n* = 911).

### Determination of covariates

Covariate selection was guided by established evidence from prior research on sarcopenia and type 2 diabetes mellitus, as well as by the availability of relevant data [17]. The covariates included in the analysis were demographic characteristics, lifestyle factors, physiological indicators, and common chronic diseases, specifically: age, gender, marital status (married and others), educational level(junior high school and below and senior high school and above), place of residence (rural, urban), smoking status(never smoked and ever smoked), drinking status, sleep conditions, depressive symptoms, hypertension, hyperlipidemia, fasting blood glucose, body mass index (BMI), glycated hemoglobin (HbA1c), triglycerides (TG), high-density lipoprotein cholesterol (HDL), low-density lipoprotein cholesterol (LDL), and common chronic comorbidities, such as cardiovascular disease.

### T2DM event assessment

The occurrence of T2DM during the follow-up period was determined using the following diagnostic criteria: (1) elevated FPG level (≥ 126 mg/dL); (2) elevated RPG level (≥ 200 mg/dL); (3) elevated HbA1c level (≥ 6.5%) [18]; and (4) self-reported physician diagnosis of diabetes [6]. In the CHARLS study, participants were instructed to undergo an overnight fast prior to venous blood sampling. Blood samples were collected by trained medical personnel and analyzed for FPG and HbA1c levels. For participants who did not meet the fasting requirement, blood samples were still obtained, and the measured glucose values were classified and analyzed as RPG.

### Statistical analysis

Continuous variables are presented as mean ± standard deviation or median (interquartile range), while categorical variables are reported as frequency (percentage). Cox proportional hazards regression models were employed to assess the associations between baseline sarcopenia status and its longitudinal changes with the risk of incident T2DM, and hazard ratios (HRs) along with their corresponding 95% confidence intervals (95% CIs) were estimated. Prior to constructing the multivariate model, we assessed multicollinearity by calculating the variance inflation factor (VIF) for all independent variables. All VIF values were below 2, indicating minimal multicollinearity and supporting the inclusion of these variables in the model. To ensure model stability and minimize the risk of overfitting, we further verified that the events per variable (EPV) ratio in the primary analytical model exceeded 20, which is well above the conventional threshold of 10, thereby supporting robust and reliable parameter estimation.

The analysis was conducted using four progressively adjusted Cox regression models. Model 0 was unadjusted. Model 1 was adjusted for age and sex. Model 2 further adjusted for marital status, residential area, and education level. Model 3 additionally included smoking status, alcohol consumption, sleep duration, depressive symptoms, BMI, HbA1c, fasting blood glucose, triglycerides, HDL, and LDL, as well as a history of hypertension, hyperlipidemia, and cardiovascular disease.

To assess the robustness of the findings, we conducted the following sensitivity analyses: (1) To minimize potential bias arising from misclassification or instability in sarcopenia status categorization, sarcopenia status was redefined using complete case data to ensure temporal consistency; (2) Random forest imputation was applied to handle missing covariate values as an alternative to multiple imputation, with the aim of evaluating the stability and reliability of the observed associations; (3)To investigate whether any level of sarcopenia is associated with the risk of diabetes, we conducted an additional sensitivity analysis by merging the “possible sarcopenia” and " sarcopenia” categories into a single group and comparing it with the “no sarcopenia” group. This analysis was designed to assess the overall effect of the sarcopenia spectrum on diabetes risk.

In addition, subgroup analyses were conducted by gender and age group (middle-aged: < 65 years; elderly: ≥ 65 years), and the significance of interaction terms was assessed using likelihood ratio tests. All statistical analyses were performed with R software (version 4.5.1). Two-sided hypothesis tests were applied, and a P value less than 0.05 was considered statistically significant. The pattern and extent of covariate missingness are summarized in Tables S1 and S2 of Supplementary File 1. Multiple imputation for missing covariates was carried out using the chained equations method, with detailed procedures provided in Supplementary File 2.

## Results

### Baseline characteristics of the study population

Our analysis included 3,673 eligible participants (mean age: 68.21 years; 51.3% male). Their baseline profiles are detailed in Table 1. Demographically, subjects with sarcopenia tended to be older, less educated, less frequently married, and more often from rural areas compared to their non-sarcopenia counterparts. Biochemically and anthropometrically, the sarcopenia group presented lower profiles of HbA1c, blood glucose, BMI, LDL, and TG, though their HDL levels exceeded those in the non-sarcopenia group.

**Table 1:** Baseline characteristics of participants for baseline sarcopenia status analyses

Characteristics	Total (*n* = 3673)	Non sarcopenia (*n* = 1331)	Possible sarcopenia (*n* = 1431)	sarcopenia (*n* = 911)	*P* value
Age, mean (SD), years	68.21 ± 6.32	66.42 ± 5.27	67.82 ± 6.03	71.44 ± 6.93	0.001
Sex, n (%)					< 0.001
female	1789(48.7)	541(40.6)	745(52.1)	503(55.2)	
male	1884(51.3)	790(59.4)	686(47.9)	408(44.8)	
Education, n (%)					< 0.001
below high school	3492(95.1)	1221(91.7)	1381(96.5)	890(97.7)	
high school and above	181(4.9)	110(8.3)	50(3.5)	21(2.3)	
Marital status, n (%)					< 0.001
Others	739(20.1)	184(13.8)	276(19.3)	279(30.6)	
Married	2934(79.9)	1147(86.2)	1155(80.7)	632(69.4)	
Residence, n (%)					0.008
Rural	3021(82.2)	1039 (78.1)	1162 (81.2)	820 (90.0)	
Urban	652 (17.8)	292 (21.9)	269 (18.8)	91 (10.0)	
Smoking status, n (%)					< 0.001
Never smokers	2075 (56.5)	708 (53.2)	842 (58.8)	525 (57.6)	
Ever smokers	1598 (43.5)	623 (46.8)	589 (41.2)	386 (42.4)	
Drinking status, n (%)					< 0.001
< One time a mounth	234 (6.4)	105 (7.9)	86 (6.0)	43 (4.7)	
>=One time a mounth	926 (25.2)	398 (29.9)	306 (21.4)	222 (24.4)	
none	2513 (68.4)	828 (62.2)	1039 (72.6)	646 (70.9)	
Sleeping status, n (%)					< 0.001
heavey	749 (20.4)	215 (16.2)	319 (22.3)	215 (23.6)	
mild	1755 (47.8)	714 (53.6)	650 (45.4)	391 (42.9)	
moderate	1169 (31.8)	402 (30.2)	462 (32.3)	305 (33.5)	
Depress, n (%)					< 0.001
heavey	474 (12.9)	126 (9.5)	200 (14.0)	148 (16.2)	
mild	1645 (44.8)	682 (51.2)	605 (42.3)	358 (39.3)	
moderate	1554 (42.3)	523 (39.3)	626 (43.7)	405 (44.5)	
HbA1c, mean (SD), %	5.11 ± 0.40	5.13 ± 0.40	5.12 ± 0.40	5.07 ± 0.39	0.001
HDL, mean (SD), mg/dl	52.62 ± 14.99	52.14 ± 15.10	49.60 ± 14.00	58.07 ± 14.87	< 0.001
BMI, mean (SD), kg/m²	22.50 ± 3.52	22.81 ± 3.31	24.31 ± 2.96	19.20 ± 1.99	< 0.001
Glucose, mean (SD), mg/dl	101.70 ± 12.20	102.06 ± 12.08	102.38 ± 12.41	100.09 ± 11.90	< 0.001
LDL, mean (SD), mg/dl	118.13 ± 33.27	119.61 ± 33.55	119.21 ± 32.98	114.25 ± 33.03	< 0.001
TG, median (IQR), mg/dl	99.12 [71.68, 139.83]	99.12 [71.68, 138.95]	107.97 [77.88, 153.99]	86.73 [66.38, 119.47]	< 0.001
Hypertension, n(%)					< 0.001
no	2712 (73.8)	1020 (76.6)	963 (67.3)	729 (80.0)	
yes	961 (26.2)	311 (23.4)	468 (32.7)	182 (20.0)	
Dyslipidemia, n (%)					0.015
no	3381 (92.1)	1218 (91.5)	1282 (89.6)	881 (96.7)	
yes	292 (7.9)	113 (8.5)	149 (10.4)	30 (3.3)	
Heart_Problems, n (%)					< 0.001
no	3172 (86.4)	1156 (86.9)	1209 (84.5)	807 (88.6)	
yes	501 (13.6)	175 (13.1)	222 (15.5)	104 (11.4)	

HbA1c, Hemoglobin A1c HDL, high-density lipoprotein cholesterol; BMI, body mass index; LDL, low-density lipoprotein cholesterol; TG, Triglycerides

The final analytical cohort for this study comprised 3,252 individuals (mean age 67.69 years; 51.2% male) who met all eligibility criteria. Table 2 summarizes the baseline characteristics of this population. To assess the robustness of the imputation procedure and the reliability of the resulting data, we compared the distributions of key variables, including age, BMI, and gender, between the imputed dataset and the original observed data. No substantial distributional differences were detected, indicating that the imputation process preserved the underlying data structure (additional File 1: Tables S3 and S4). The analytical results derived from the imputed data were consistent with the patterns observed in Tables 1 and 2, further supporting the validity of our findings.

**Table 2:** Baseline characteristics of participants for changes in sarcopenia status analyses

Characteristics	Total (*n* = 3252)	Non sarcopenia (*n* = 1220)	Possible sarcopenia (*n* = 1278)	sarcopenia (*n* = 754)	*P* value
Age, mean (SD), years	67.69 ± 5.96	66.18 ± 5.10	67.47 ± 5.83	70.50 ± 6.47	< 0.001
Sex, n (%)					< 0.001
female	1587 (48.8)	496 (40.7)	664 (52.0)	427 (56.6)	
male	1665 (51.2)	724 (59.3)	614 (48.0)	327 (43.4)	
Education, n (%)					< 0.001
below high school	3099 (95.3)	1123 (92.0)	1237 (96.8)	739 (98.0)	
high school and above	153 (4.7)	97 (8.0)	41 (3.2)	15 (2.0)	
Marital status, n (%)					< 0.001
Others	615 (18.9)	160 (13.1)	239 (18.7)	216 (28.6)	
Married	2637 (81.1)	1060 (86.9)	1039 (81.3)	538 (71.4)	
Residence, n (%)					< 0.001
Rural	2705 (83.2)	968 (79.3)	1050 (82.2)	687 (91.1)	
Urban	547 (16.8)	252 (20.7)	228 (17.8)	67 (8.9)	
Smoking status, n (%)					0.024
Never smokers	1856 (57.1)	659 (54.0)	754 (59.0)	443 (58.8)	
Ever smokers	1396 (42.9)	561 (46.0)	524 (41.0)	311 (41.2)	
Drinking status, n (%)					< 0.001
< One time a mounth	213 (6.5)	99 (8.1)	78 (6.1)	36 (4.8)	
>=One time a mounth	826 (25.4)	366 (30.0)	279 (21.8)	181 (24.0)	
none	2213 (68.1)	755 (61.9)	921 (72.1)	537 (71.2)	
Sleeping status, n (%)					< 0.001
heavey	657 (20.2)	195 (16.0)	282 (22.1)	180 (23.9)	
mild	1552 (47.7)	649 (53.2)	580 (45.4)	323 (42.8)	
moderate	1043 (32.1)	376 (30.8)	416 (32.6)	251 (33.3)	
Depress, n (%)					< 0.001
heavey	407 (12.5)	117 (9.6)	171 (13.4)	119 (15.8)	
mild	1473 (45.3)	623 (51.1)	550 (43.0)	300 (39.8)	
moderate	1372 (42.2)	480 (39.3)	557 (43.6)	335 (44.4)	
HbA1c, mean (SD), %	5.13 ± 0.40	5.14 ± 0.40)	5.14 ± 0.39)	5.09 ± 0.39)	0.006
HDL, mean (SD), mg/dl	53.07 ± 15.31	52.44 ± 15.31	50.04 ± 14.11	59.22 ± 15.50	< 0.001
BMI, mean (SD), kg/m²	22.55 ± 3.45	22.79 ± 3.24	24.29 ± 2.96	19.21 ± 1.81	< 0.001
LDL, mean (SD), mg/dl	117.96 ± 33.38	118.65 ± 33.02	118.78 ± 33.50	115.47 ± 33.68	0.064
TG, median (IQR), mg/dl	99.12 [71.68, 139.83]	100.00 [72.57, 138.95]	106.64 [77.22, 153.10]	85.85 [66.38, 120.36]	< 0.001
Glucose, median (IQR), mg/dl	101.34 [94.14, 109.26]	101.88 [94.68, 109.44]	101.70 [94.68, 109.98]	100.08 [92.21, 107.64]	< 0.001
Hypertension, n(%)					< 0.001
no	2416 (74.3)	935 (76.6)	868 (67.9)	613 (81.3)	
yes	836 (25.7)	285 (23.4)	410 (32.1)	141 (18.7)	
Dyslipidemia, n (%)					< 0.001
no	2997 (92.2)	1119 (91.7)	1150 (90.0)	728 (96.6)	
yes	255 (7.8)	101 (8.3)	128 (10.0)	26 (3.4)	
Heart_Problems, n (%)					0.096
no	2815 (86.6)	1060 (86.9)	1088 (85.1)	667 (88.5)	
yes	437 (13.4)	160 (13.1)	190 (14.9)	87 (11.5)	

HbA1c, Hemoglobin A1c HDL, high-density lipoprotein cholesterol; BMI, body mass index; LDL, low-density lipoprotein cholesterol; TG, Triglycerides

**Fig. 2:**
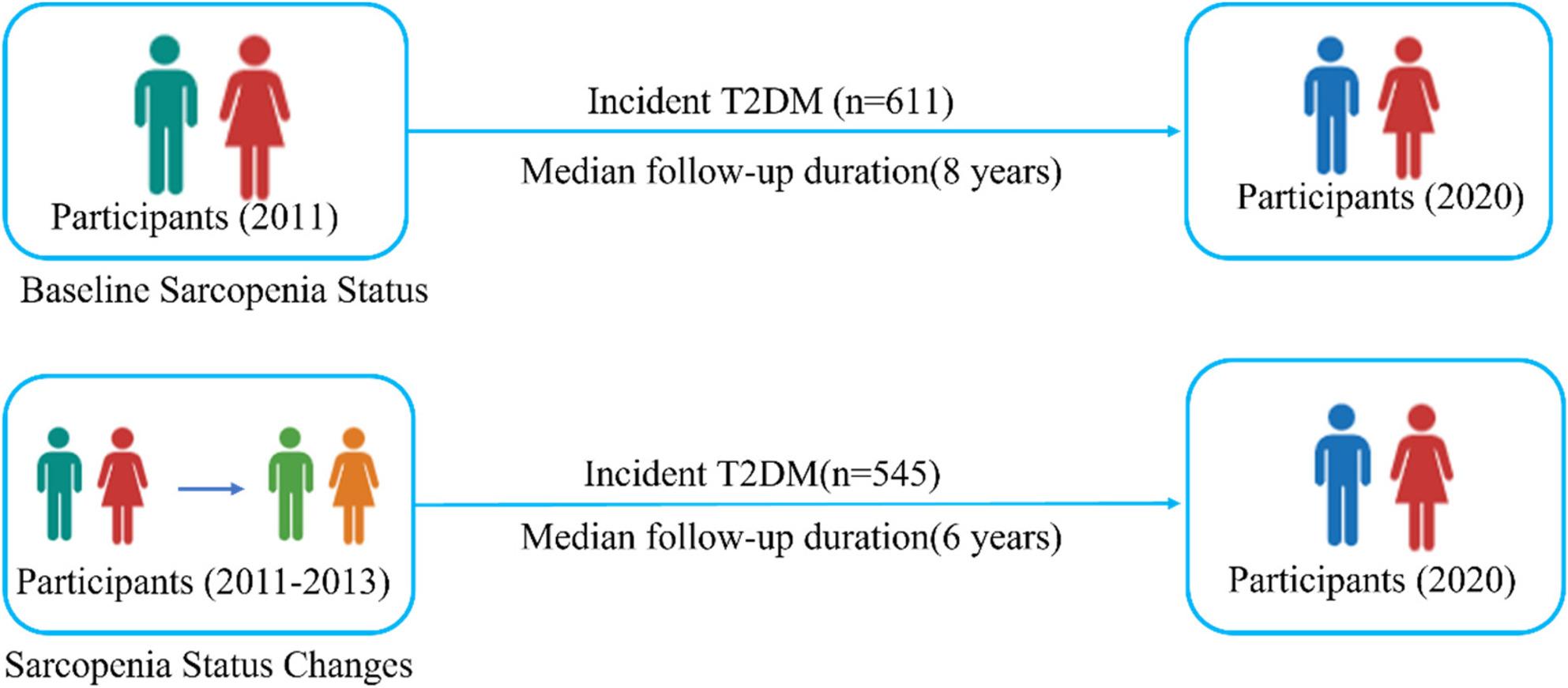
CHARLS Cohort Analysis Overview

With a median follow-up of 8 years, the analysis of baseline sarcopenia status in the CHARLS cohort identified 611 participants who developed incident T2DM. The analysis of sarcopenia status changes, which had a median follow-up of 6 years, found 545 incident T2DM cases (Fig. 2).

### Association of baseline sarcopenia status with incident T2DM

As summarized in Supplementary File 1: Table S5, baseline sarcopenia status showed a variable correlation with T2DM incidence. After adjustment for confounders, the hazard ratio for possible sarcopenia was 1.23 (95% CI 1.01–1.50), indicating a significantly higher risk than the no sarcopenia group. A similarly elevated but non-significant HR of 1.13 (95% CI 0.85–1.50) was found for confirmed sarcopenia, which lacked statistical significance. After merging the “possible sarcopenia” and “confirmed sarcopenia” categories into a single group, the combined group exhibited a significantly higher risk of diabetes compared to the non-sarcopenia group (HR = 1.22, 95% CI: 1.01–1.47, *p* = 0.039). These findings indicate that even early-stage sarcopenia may be associated with an increased risk of developing diabetes (additional File 1: Tables S9).

### Association of changes in sarcopenia status with incident T2DM

**Fig. 3:**
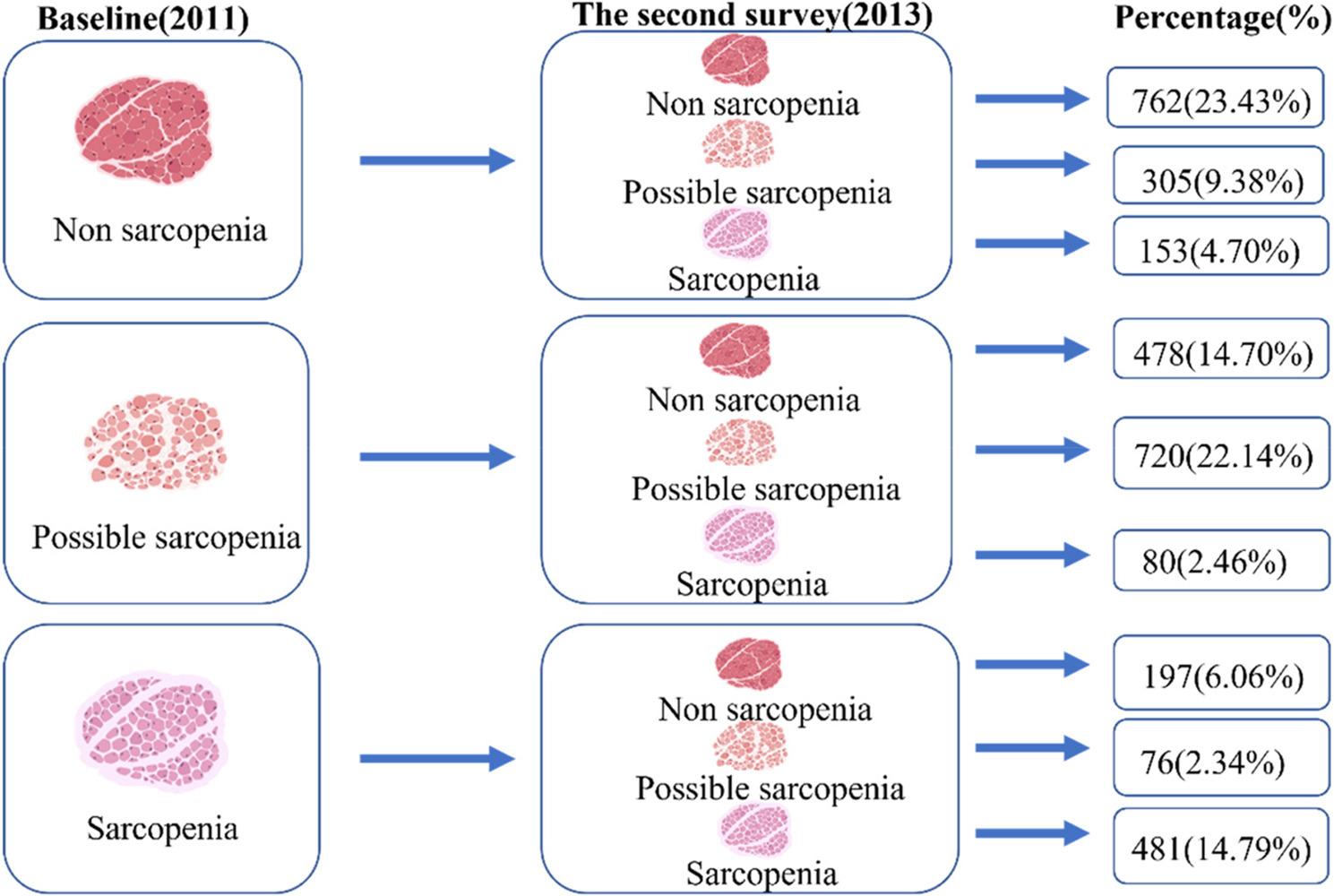
Participant Distribution by Sarcopenia Status Change (2011–2013)

Figure 2 presents the number and percentage of participants who experienced a change in sarcopenia status during the 2-year follow-up period. Among individuals without sarcopenia at baseline, 458 (37.5%) progressed to either possible sarcopenia or confirmed sarcopenia. Conversely, among those with baseline sarcopenia, 273 (36.2%) reverted to a non-sarcopenic or possible sarcopenic state (Fig. 3).

Table 3 explores the association between dynamic changes in sarcopenia status and incident T2DM. Adjusted analyses indicated a significant protective effect for individuals who transitioned out of sarcopenia, with an HR of 0.61 (95% CI 0.40–0.93). In contrast, progression to possible or confirmed sarcopenia was associated with a non-significant elevation in T2DM risk.

**Table 3:** Association of changes in sarcopenia status with risks of incident T2DM

		**Crude model**	**Model 1**	**Model 2**	**Model 3**
Character	Events/n	HR (95%CI)	HR (95%CI)	HR (95%CI)	HR (95%CI)
Stable non-sarcopenia	115/762	Reference	Reference	Reference	Reference
Non-sarcopenia to possible sarcopenia	58/305	0.93 (0.66,1.30)	0.91 (0.64,1.29)	0.93 (0.66,1.31)	1.18 (0.81,1.72)
Non-sarcopenia to sarcopenia	15/153	0.90 (0.67,1.22)	0.90 (0.67,1.22)	0.90 (0.67,1.22)	1.03 (0.76,1.40)
Possible sarcopenia to non-sarcopenia	88/478	0.97 (0.67,1.41)	0.97 (0.67,1.42)	0.97 (0.66,1.42)	1.02 (0.70,1.48)
Stable possible sarcopenia	159/720	1.10 (0.76,1.59)	1.10 (0.76,1.59)	1.10 (0.76,1.58)	0.94 (0.65,1.38)
Possible sarcopenia to sarcopenia	10/80	0.93 (0.60,1.43)	0.92 (0.60,1.41)	0.92 (0.60,1.41)	0.89 (0.58,1.37)
Sarcopenia to non-sarcopenia	25/197	0.50 (0.33,0.74)	0.50 (0.33,0.74)	0.50 (0.33,0.74)	0.61 (0.40,0.93)
Sarcopenia to possible sarcopenia	13/76	1.37 (0.87,2.16)	1.40 (0.89,2.21)	1.39 (0.88,2.19)	1.23 (0.77,1.96)
Stable sarcopenia	62/481	1.08 (0.74,1.57)	1.08 (0.74,1.58)	1.08 (0.74,1.58)	1.07 (0.73,1.56)

Model 1 included adjustments for age and gender; Model 2 further adjusted for marriage, residence, and education level; and Model 3 additionally adjusted for drink, sleep status, depress, hypertension, dyslipidemia, heart problems, Glucose, TG, HbA1c, HDL, LDL, BMI; HbA1c, Hemoglobin A1c; TG, triglyceride-glucose; HDL, high-density lipoprotein cholesterol; LDL, low-density lipoprotein cholesterol; BMI, body mass index

### Subgroup analyses and sensitivity analyses

In the subgroup analysis, men who transitioned from sarcopenia to a non-sarcopenic state exhibited a significantly lower risk of developing incident T2DM (HR 0.43, 95% CI 0.24–0.78). However, this association was not statistically significant among female participants. Furthermore, among individuals aged 65 years and older, those who recovered from sarcopenia to non-sarcopenia showed a significantly reduced risk of incident T2DM (HR 0.60, 95% CI 0.37–0.99), whereas no significant risk reduction was observed in individuals under 65 years of age. Detailed results of the diabetes-related subgroup analyses are presented in Supplementary File 1: Tables S8.

To further evaluate the robustness of our findings, we performed a complete case analysis (CCA) by excluding participants with missing data in the outcome, exposure, or any covariate variables. This analysis included 2,344 individuals, representing 72.07% of the original sample. The objective was to assess the association between changes in sarcopenia status and the risk of incident T2DM under conditions where all key variables were fully observed. The results remained consistent, showing that recovery from sarcopenia to a non-sarcopenic state was significantly associated with a lower risk of developing T2DM (Additional File 1: Table S6). Furthermore, when missing data were re-imputed using the random forest imputation method, the observed associations remained stable and statistically consistent (Additional File 1: Table S7).

## Discussion

In this prospective cohort study, we investigated the associations of baseline sarcopenia status and its dynamic changes with the risk of incident T2DM. Our results indicated that individuals with possible sarcopenia had a significantly elevated risk of T2DM compared to those without sarcopenia. Although an increased risk was also observed among participants with confirmed sarcopenia, this association was not statistically significant after full adjustment for potential confounders. Although no significant association was observed between confirmed sarcopenia and the risk of diabetes, the combined analysis of possible and confirmed sarcopenia revealed a statistically significant increase in diabetes risk. This finding can be interpreted through several perspectives. First, sarcopenia is a continuous and progressive condition, in which even early-stage reductions in muscle mass and function, though subclinical, may already disrupt glucose metabolic homeostasis [19]. Second, pooling the two groups increased the sample size, thereby enhancing statistical power to detect modest but meaningful associations. Finally, this result suggests that sarcopenia-related traits may serve as early biological markers for diabetes risk, offering potential clinical utility even before individuals meet full diagnostic criteria for sarcopenia.Moreover, those who transitioned from a sarcopenic to a non-sarcopenic state during follow-up showed a significantly lower risk of developing T2DM.

Against the backdrop of global aging, the prevalence of geriatric syndromes such as sarcopenia continues to rise. Accumulating evidence supports a bidirectional relationship between sarcopenia and diabetes [20, 21]. A prospective cohort study among middle-aged and older adults demonstrated that, during a 4-year follow-up, lower grip strength (OR: 2.31, 95% CI: 1.74–3.08) and reduced appendicular skeletal muscle mass index (ASM/Ht²) (OR: 1.25, 95% CI: 1.20–1.30) were significantly associated with an increased risk of diabetes. Conversely, elevated fasting plasma glucose (OR: 1.52, 95% CI: 1.17–1.96) and higher glycated hemoglobin levels (OR: 1.35, 95% CI: 1.05–1.73) were also independently linked to a greater risk of sarcopenia [7]. A meta-analysis further indicated that the prevalence of sarcopenia among individuals with diabetes is approximately threefold higher than in those without diabetes and is associated with worse clinical outcomes, indicating a positive association between the two conditions [22]. Data from the 2009–2010 Korea National Health and Nutrition Examination Survey (KNHANES) also identified sarcopenia as a significant risk factor for diabetes in non-obese individuals (OR: 2.140; 95% CI: 1.549–2.956; *P* < 0.001) [23], and Luo et al. reported a potential association between the two conditions in the general population [6].

Our findings are consistent with this body of evidence in showing that possible sarcopenia is associated with a higher risk of T2DM in a prospective setting. However, it is important to note that the association for confirmed sarcopenia was not statistically significant after multivariate adjustment, suggesting that the relationship may be influenced by confounding factors or vary by sarcopenia severity. Therefore, while our study supports the role of possible sarcopenia as a potential risk marker, the evidence regarding confirmed sarcopenia remains less clear.

A distinctive aspect of our study is the focus on changes in sarcopenia status, a dimension that has been seldom examined in previous research. Existing studies have highlighted the dynamic nature of sarcopenia in older Chinese adults [24]. For instance, one study of 4,822 participants found that 19.1% of those without baseline sarcopenia progressed to possible or confirmed sarcopenia during follow-up, while among those with baseline sarcopenia, 49.0% reverted to a non-sarcopenic or possible sarcopenic state, indicating a potential for recovery [11]. Our analysis corroborates the fluctuating nature of sarcopenia in the CHARLS cohort. More importantly, we found that participants who recovered from sarcopenia to a non-sarcopenic state had a significantly lower risk of T2DM compared to those who remained non-sarcopenic throughout the study. This suggests that improvement in sarcopenia status may help reduce diabetes risk. Sensitivity analyses using complete-case data and alternative imputation methods yielded consistent results, supporting the robustness of this finding. Nevertheless, further validation in independent cohorts is needed to confirm its generalizability.

Several mechanisms may explain how changes in sarcopenia status influence T2DM risk. Sarcopenia and T2DM share common pathophysiological pathways, with insulin resistance serving as a central link [25]. Insulin resistance impairs glucose homeostasis and disrupts muscle protein metabolism, contributing to reduced muscle mass and strength. It may also affect the PI3K/Akt/mTOR signaling pathway, which regulates protein synthesis and muscle growth. In T2DM, insulin resistance can suppress Akt and mTOR activity, thereby inhibiting protein synthesis, while promoting the activation of FoxO transcription factors and the ubiquitin-proteasome system, leading to muscle atrophy [26, 27]. Skeletal muscle insulin resistance is also closely related to oxidative stress and chronic low-grade inflammation. Pro-inflammatory cytokines such as TNF-α, IL-6, and IL-1 can exacerbate insulin resistance and promote muscle degeneration via NF-κB activation [28]. Concurrently, oxidative stress may damage DNA, impair muscle repair, and compromise satellite cell function [29],, creating a vicious cycle that worsens both sarcopenia and diabetes. In contrast, improvements in muscle mass have been associated with enhanced insulin sensitivity [30]. Given that skeletal muscle accounts for approximately 80% of insulin-stimulated glucose disposal [31], changes in muscle status may directly affect systemic glucose regulation. Together, these mechanisms suggest that dynamic changes in sarcopenia could play a role in T2DM development.

Conversely, the findings of this study suggest that enhancing skeletal muscle mass and function may represent a promising therapeutic target for the prevention of type 2 diabetes mellitus (T2DM), owing to the reversibility of the underlying pathophysiological mechanisms. First, increased muscle mass directly improves insulin-mediated glucose disposal by expanding both the capacity and efficiency of glucose uptake. Second, improvements in muscle quality are associated with enhanced endocrine activity: contracting myofibers release higher levels of beneficial myokines, such as irisin, which promote adipose tissue thermogenesis and hepatic glycogen synthesis, while expression of detrimental regulators like myostatin is reduced, leading to systemic enhancement of insulin sensitivity [32]. At the molecular level, these adaptations involve activation of the PI3K/Akt/mTOR anabolic signaling pathway and concurrent suppression of the FoxO/ubiquitin-proteasome proteolytic system. Thus, skeletal muscle is not merely a passive “victim” of insulin resistance but also an active “regulator” of whole-body metabolic homeostasis. Dynamic shifts in muscle status, whether loss or accretion, are mediated through shared, bidirectional physiological and molecular pathways that directly and precisely modulate glucose metabolism. These mechanistic insights provide a robust pathophysiological foundation for interventions such as resistance training and nutritional support aimed at improving muscle health to reduce T2DM risk.

This study has several clinical and public health implications. First, assessing sarcopenia, especially in its early or possible stage, may help identify older adults at high risk of T2DM. Second, the observed association between sarcopenia reversal and reduced diabetes risk highlights the potential value of interventions aimed at improving muscle health. Possible sarcopenia, which is simple to assess in clinical practice, could serve as a practical early warning indicator. Future studies, including randomized trials, are needed to identify effective strategies for sarcopenia management and reversal in the context of diabetes prevention.

The research also exhibits several notable strengths. To our knowledge, it is the first to examine the relationship between dynamic sarcopenia transitions and T2DM incidence. The use of a large, nationally representative cohort enhances the generalizability of findings to middle-aged and older adults in China. Multiple sensitivity analyses further strengthen the reliability of the results.

Several limitations in the research require careful consideration. First, the diagnosis of diabetes was based on a combination of fasting blood glucose, glycated hemoglobin, self-reported physician diagnosis, and the use of antihyperglycemic medications [33]. Blood samples were collected only at baseline and the third follow-up; consequently, incident cases occurring between these time points were primarily identified through self-report, without confirmation from medical records or continuous biomarker monitoring. This approach may introduce classification misclassification bias, although prior studies have demonstrated relatively high accuracy of self-reported diabetes in certain populations [34, 35]. Given that undetected cases (false negatives) are likely more prevalent than incorrectly classified individuals without disease (false positives), such non-differential misclassification tends to attenuate the observed association, thereby resulting in a conservative bias and an underestimation of the true risk.Second, although multiple known confounders were adjusted for in the statistical models, residual confounding remains possible due to the inherent nature of observational designs. Unmeasured or incompletely assessed factors, such as family history of diabetes, genetic predisposition, detailed dietary patterns, and nutritional status, may independently influence both sarcopenia and T2DM risk, potentially biasing the observed associations. Third, appendicular skeletal muscle mass was estimated using prediction equations rather than directly measured by dual-energy X-ray absorptiometry, which may result in measurement bias. Compared to DXA, this estimation method is unable to differentiate between lean muscle mass and intramuscular fat infiltration. This form of measurement error is non-differential and tends to bias the effect size toward the null, resulting in an underestimation of the true association. Fourth, one important limitation of this study is the inability to fully exclude the possibility of reverse causality. Although individuals with diagnosed diabetes at baseline and the first follow-up were excluded, and a temporal sequence was observed in which changes in sarcopenia status preceded the onset of diabetes, undetected subclinical hyperglycemia or insulin resistance may have been present during the observation period. Such early metabolic disturbances could concurrently accelerate the decline in muscle mass and function, thereby contributing to sarcopenia progression, and increase the likelihood of subsequent diabetes diagnosis. Thus, these underlying metabolic abnormalities may represent a shared pathophysiological mechanism linking sarcopenia and diabetes, rather than indicating that sarcopenia per se directly causes diabetes. Finally, to ensure data completeness and analytical reliability, participants with missing values in key variables were excluded. While this enhances internal data quality, it may introduce selection bias if the excluded individuals differ systematically from those included in terms of demographic or health-related characteristics, thereby affecting the generalizability of the findings.These methodological considerations highlight important avenues for future research, such as employing more precise muscle mass assessment techniques and implementing more frequent biological assessments to improve the ascertainment of incident diabetes. We contend that, with careful acknowledgment and interpretation of these limitations, this study provides meaningful epidemiological evidence regarding the association between dynamic changes in sarcopenia status and the risk of T2DM.

## Conclusions

This prospective study demonstrates that dynamic changes in sarcopenia status are associated with the risk of developing type 2 diabetes mellitus (T2DM). Specifically, transitioning from a sarcopenic to a non-sarcopenic state is significantly linked to a lower risk of incident T2DM. These findings suggest that reversing sarcopenia may represent a promising and modifiable target for the prevention of type 2 diabetes. Future research should prioritize the development and implementation of targeted interventions, such as structured programs combining resistance exercise and nutritional support, designed to reverse sarcopenia, and evaluate their causal efficacy and public health impact in reducing T2DM incidence through rigorously designed randomized controlled trials.

## Supplementary Information

Supplementary Material 1.

Supplementary Material 2.

## Data Availability

The datasets analyzed during the current study are available from the CHARLS project at Peking University, http://charls.pku.edu.cn/.

## Abbreviations

T2DM: Type 2 diabetes mellitus
CHARLS: China Health and Retirement Longitudinal Study
AWGS: Asian Working Group for Sarcopenia
FPG: fasting plasma glucose
RPG: random plasma glucose
HbA1c: Glycated emoglobin
HRs: Hazard ratios
CIs: Confidence intervals
ASM: Appendicular Skeletal muscle mass
DXA: X-ray absorptiometry
HDL: High-density lipoprotein cholesterol
LDL: Low-density lipoprotein cholesterol
TG: Triglycerides
FPG: Fasting plasma glucose
BMI: Body Mass Index
RPG: Random plasma glucose
HR: Hazard Ratios
CCA: Complete Case Analysis
KNHANES: Korean National Health and Nutrition Examination Survey
PI3: phosphatidylinositol-3
AMPK: AMP-activated protein kinase
GPs: General practitioners
