# Supplementary material for: Sarcopenia changes and incident type 2 diabetes mellitus: a prospective analysis of the CHARLS cohort
Source: BMC Geriatr. 2026 Mar 13;26:556. doi: 10.1186/s12877-026-07169-4 (PMC13097902; doi:10.1186/s12877-026-07169-4)
Supplement: Supplementary file 1 — Supplementary Material 1. [file 12877_2026_7169_MOESM1_ESM.docx]

**Supplemental Methods**

**Multiple imputation**

Multiple imputation was employed to address missing data among covariates, specifically using the multiple imputation by chained equations (MICE) approach. In the CHARLS dataset, imputation was restricted to covariates with a missing rate below 80%, as recommended by prior research. All eligible covariates were imputed using a model that included the following variables: education level, residential address, smoking status, drinking status, sleep quality, depressive symptoms, hypertension, hyperlipidemia, heart disease, body mass index (BMI), glycated hemoglobin (HbA1c), high-density lipoprotein cholesterol (HDL), low-density lipoprotein cholesterol (LDL), blood glucose, and triglycerides. Within each cohort, five imputations were performed, resulting in five imputed datasets. Effect estimates were calculated separately for each dataset and subsequently combined using Rubin’s rules to account for between- and within-imputation variability. Multiple imputation was conducted using the R package "mice".
